# Peripheral Fluorescein Angiographic Findings in Fellow Eyes of Patients with Branch Retinal Vein Occlusion

**DOI:** 10.1155/2013/464127

**Published:** 2013-03-31

**Authors:** Irena Tsui, Asima Bajwa, Valentina Franco-Cardenas, Carolyn K. Pan, Hanna Y. Kim, Steven D. Schwartz

**Affiliations:** Retina Division, Jules Stein Eye Institute, University of California, 100 Stein Plaza, Los Angeles, CA 90095, USA

## Abstract

*Introduction*. Branch retinal vein occlusion (BRVO) is a common retinal vascular condition that results in intraocular inflammatory changes. Ultra wide field fluorescein angiography (UWFFA) is a retinal imaging device that can capture peripheral retinal findings. The purpose of this study was to look for peripheral findings in the fellow eye of patients with BRVO using UWFFA. *Methods*. Retrospective imaging review of patients diagnosed with BRVO that had both eyes imaged with UWFFA. Images were graded for peripheral findings in other quadrants of the same eye as well as in all quadrants of the fellow eye. *Results*. Of 81 patients, 14 (17%) patients had late vascular leakage in a quadrant other than the BRVO distribution. Five (6%) findings were in the same eye, 8 (10%) findings were in the fellow eye, and 1 (1%) finding was in both the same eye and the fellow eye. Of these 14 patients, 11 (80%) patients had hypertension. *Conclusion*. Late peripheral retinal leakage in the fellow eye of patients with BRVO was detected in this cohort of patients with UWFFA. This novel finding may represent underlying systemic inflammation, hypertension, or bilateral BRVOs.

## 1. Introduction

Branch retinal vein occlusion (BRVO) affects approximately 1% of the population and can cause severe vision loss through macular edema, retinal neovascularization, and retinal detachment [1–4]. The disease is estimated to be bilateral in 5% of patients at presentation and become bilateral in 15% of patients over time [3, 5]. When bilateral and/or multiple, systemic vasculitis such as sarcoidosis, systemic lupus erythematosus, or Behcet's disease may be the underlying etiology. 

Atherosclerosis risk factors such as hypertension and hypercholesterolemia are thought to contribute to BRVO formation by causing arterial wall hardening and inflammation at arteriovenous crossing sites [6]. In theory, systemic risk factors should put patients at similar risk for BRVO in both eyes and it is unclear why the disease is typically unilateral. One explanation could be due to random and individual variation in arteriovenous crossing patterns [7].

There are several studies examining the vitreous of eyes that have suffered BRVO showing that there are increased inflammatory mediators when compared with control surgical patients. For example, vascular endothelial growth factor (VEGF), soluble intercellular adhesion molecule-1, interleukin-8, and interleukin-6 were among the inflammatory markers found to be elevated in the vitreous of BRVO eyes [8–10]. In these studies inflammatory markers were correlated to macular edema [10] and the size of BRVO [8]. However, vitreous samples were not taken before BRVO was detected nor was the vitreous of the fellow eye assessed as vitreous removal is invasive and there was no clinical indication. Therefore, it is not certain if the increase in inflammatory markers was a cause or effect of the disease.

Ultra wide field fluorescein angiography (UWFFA, Optos, Marlborough, MA, USA) is a retinal imaging technology that captures up to 200 degrees of the retina in a single picture and has been useful in detecting peripheral findings in a variety of retinal vascular diseases including diabetes, vein occlusions, and uveitis [11–13]. Prior work by our group studying peripheral retinal findings in BRVO using UWFFA found that peripheral nonperfusion and vascular leakage were detectable [11]. In a cohort of 80 patients, peripheral nonperfusion was associated with angiographic macular edema, while vascular leakage was not. The purpose of this current study was to assess peripheral findings on fluorescein angiography in other quadrants of the same eye and in fellow eyes of patients with BRVO.

## 2. Materials and Methods

This retrospective imaging study was Institutional Review Board approved and carried out at the Jules Stein Eye Institute in Los Angles, CA, USA. An imaging database was searched for patients with the diagnosis of BRVO who underwent UWFFA (Optos, Marlborough, MA, USA). Patients with another diagnosis or with concurrent other retinal diseases (i.e., age-related macular degeneration) were excluded. Patients with poorly controlled diabetes or signs of diabetic retinopathy such as intraretinal hemorrhage and venous beading were excluded. Patients with known inflammatory disorders such as sarcoidosis, systemic lupus erythematosus, and Behcet's disease were excluded.

Demographic data such as patient gender and age were collected. Significant past medical history such as hypertension, high cholesterol, and diabetes mellitus were noted. All images were reviewed with Vantage Review Software (Optos, Marlborough, MA, USA) and adjusted with zoom, gamma, and contrast to optimize image quality. The quadrant affected by a BRVO was recorded. Other quadrants of the same eye were assessed for peripheral findings. Similarly, all quadrants of the fellow eye were graded for peripheral findings.

## 3. Results 

A total of 84 patients with BRVO were included in this study. Forty-nine (58%) patients were female and 55 (65%) eyes were OD. Average patient age was 60 years old (range 34–94 years; SD 13 years). Sixteen (19%) patients had no significant past medical history. Thirty-eight (45%) patients had hypertension only. Nineteen (23%) patients had hypertension and high cholesterol only. Five (6%) patients had hypertension and diabetes mellitus only. Four (5%) patients had hypertension, high cholesterol, and diabetes mellitus. Two (2%) patients had diabetes mellitus only.

Thirteen (15%) BRVOs were macular and 71 (85%) were quadrantic. Of the macular BRVOs, 10 (77%) were superior and 3 (23%) were inferior. Of the quadrantic BRVOs, 40 (56%) were superotemporal, 25 (35%) were inferotemporal, 3 (4%) were inferonasal, and 1 (1%) was superonasal. Two (3%) patients had two quadrantic BRVOs in the same eye.

Three (4%) patients were known to have bilateral BRVOs at the time of presentation. Of the remaining 81 patients, 14 (17%) patients had late vascular leakage in a quadrant other than the BRVO distribution. Five (6%) findings were in the same eye, 8 (10%) findings were in the fellow eye, and 1 (1%) finding was in both the same eye and the fellow eye. The six eyes (7%) with leakage in the same eye all had leakage in a quadrant contiguous to the BRVO. Of the 14 patients with leakage in a quadrant other than the BRVO, 8 (57%) patients had hypertension only; 2 (14%) patients had hypertension, high cholesterol, and diabetes mellitus; 1 (7%) patient had hypertension and diabetes mellitus only; 1 (7%) patient had high cholesterol only; and 2 (14%) patients had no known systemic diseases. 

## 4. Discussion

**Figure 1:**
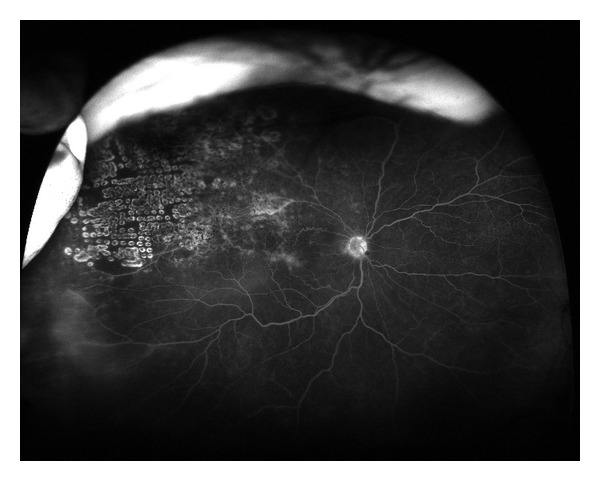
Ultra wide field fluorescein angiogram of an eye with a superotemporal branch retinal vein occlusion. There is also late peripheral leakage in the inferotemporal quadrant.

**Figure 2:**
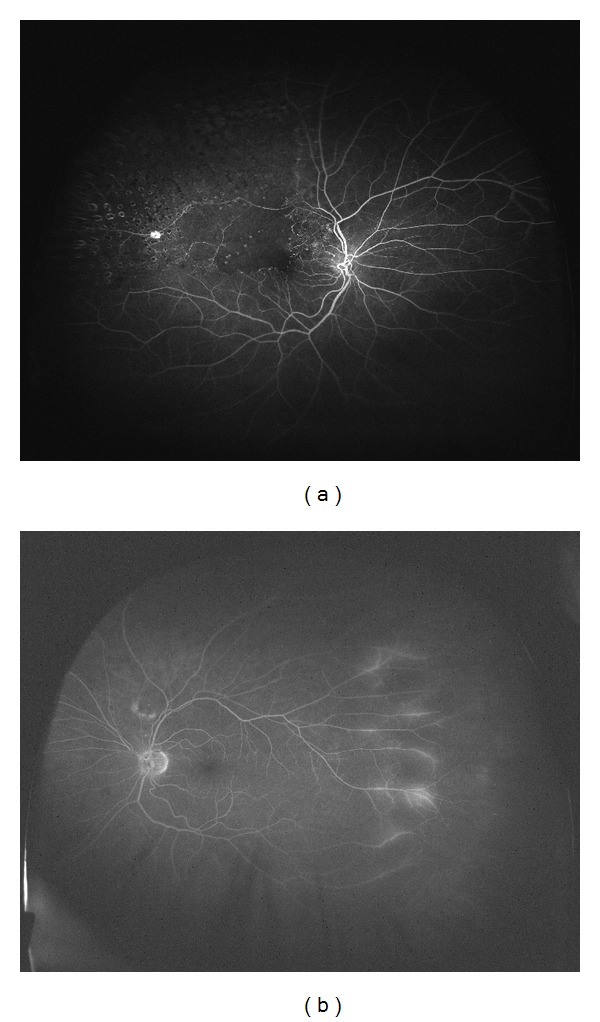
(a) Ultra wide field angiogram of a patient with branch retinal vein occlusion in the right eye. (b) The fellow eye has late peripheral leakage temporally.

This study examined peripheral retinal changes in patients with BRVO in other quadrants of the same eye and in the fellow eye using UWFFA. In 81 patients, 14 (17%) patients had late vascular leakage in a quadrant other than the BRVO distribution. Two examples from our study are shown in Figures 1 and 2. 

There are three explanations for detecting late peripheral leakage in 11% (*n* = 9) of fellow eyes. One possibility is that inflammation is not only a consequence of vein occlusion, but it is also a part of the pathogenesis of BRVO and patients with BRVO have higher levels of inflammation systemically. This hypothesis has been tested by examining gene polymorphisms related to inflammatory cytokines in BRVO patients and normal control patients [14]. In a study of almost 400 patients, 10 single nucleotide polymorphisms were assayed but were not found to be independent risk factors for BRVO suggesting that inflammation did not contribute to the pathogenesis of BRVO. 

A multiethnic epidemiological study of over 6000 subjects considered the association of retinal vein occlusion (RVO) to traditional cardiovascular risk factors [1]. RVO was not associated with systemic inflammation, hematological abnormalities, or atherosclerosis but it was associated with hypertension and dyslipidemia. Given the high prevalence of hypertension in our group of patients, a second explanation for late peripheral leakage in the fellow eye is that it was a manifestation of hypertension. 

A third explanation of bilateral leakage is that BRVO may be underrecognized as a bilateral disease and these areas of late peripheral leakage are actually small peripheral BRVOs that are otherwise undetectable with clinical exam and traditional imaging. This would be supported by the fact that the distribution of leakage was often in a venous distribution. Without coexisting macular edema or peripheral ischemia, small incidentally found BRVOs do not require treatment but the finding does warrant further observation to detect progression of disease.

Lastly, it is possible that these small areas of peripheral vascular leakage are not pathological and may be found in the general population. To our knowledge, there is no prior study establishing UWFFA findings in healthy patients without known ocular or systemic disease. In 1985, a study using traditional fluorescein angiography in 25 patients did not detect peripheral leakage in normal patients [15]. However, this was before UWFFA was available and likely incompletely captured the periphery.

Limitations of our study are related to its retrospective nature, sample size, and lack of a control group. Some patients had anti-VEGF treatment within two months of having UWFFA and this may have masked leakage in the treated eye. It is possible that patients with unexpected peripheral leakage do in fact have a coexisting systemic illness that was not documented in the chart. Furthermore, patients at a tertiary referral center may not represent the general population. Nonetheless, a subset of patients were found to have unexpected late peripheral leakage on UWFFA of possible clinical significance.

In conclusion, 11% of patients with BRVO in our population had unexpected late peripheral leakage in fellow eyes on UWFFA. It may represent an underlying inflammatory condition, hypertensive changes, or bilateral BRVO. Further studies to evaluate a larger population of BRVO patients in a protocol manner should be considered to further elucidate the significance of these findings.
